# Origin of extremely large magnetoresistance in the candidate type-II Weyl semimetal MoTe_**2−*x***_

**DOI:** 10.1038/s41598-018-32387-1

**Published:** 2018-09-17

**Authors:** Sangyun Lee, Jaekyung Jang, Sung-Il Kim, Soon-Gil Jung, Jihyun Kim, Suyeon Cho, Sung Wng Kim, Joo Yull Rhee, Kee-Su Park, Tuson Park

**Affiliations:** 1 Center for Quantum Materials and Superconductivity (CQMS) and Department of Physics, Sungkyunkwan University, Suwon, 16419 South Korea; 2 Department of Physics, Sungkyunkwan University, Suwon, 16419 South Korea; 3 Center for Integrated Nanostructure Physics (CINAP), Institute for Basic Science (IBS), Sungkyunkwan University, Suwon, 16419 South Korea; 4 Division of Chemical Engineering and Materials Science, Ewha Womans University, Seoul, 03760 South Korea; 5 Department of Energy Science, Sungkyunkwan University, Suwon, 16419 South Korea

## Abstract

The recent observation of extremely large magnetoresistance (MR) in the transition-metal dichalcogenide MoTe_2_ has attracted considerable interest due to its potential technological applications as well as its relationship with novel electronic states predicted for a candidate type-II Weyl semimetal. In order to understand the origin of the MR, the electronic structure of MoTe_2−*x*_ (*x* = 0.08) is systematically tuned by application of pressure and probed via its Hall and longitudinal conductivities. With increasing pressure, a monoclinic-to-orthorhombic (1 T′ to T_d_) structural phase transition temperature (T*) gradually decreases from 210 K at 1 bar to 58 K at 1.1 GPa, and there is no anomaly associated with the phase transition at 1.4 GPa, indicating that a *T* = 0 K quantum phase transition occurs at a critical pressure (*P*_*c*_) between 1.1 and 1.4 GPa. The large MR observed at 1 bar is suppressed with increasing pressure and is almost saturated at 100% for *P* > *P*_*c*_. The dependence on magnetic field of the Hall and longitudinal conductivities of MoTe_2−*x*_ shows that a pair of electron and hole bands are important in the low-pressure T_d_ phase, while another pair of electron and hole bands are additionally required in the high-pressure 1 T′ phase. The MR peaks at a characteristic hole-to-electron concentration ratio (*n*_*c*_) and is sharply suppressed when the ratio deviates from *n*_*c*_ within the T_d_ phase. These results establish the comprehensive temperature-pressure phase diagram of MoTe_2−*x*_ and underscore that its MR originates from balanced electron-hole carrier concentrations.

## Introduction

Transition-metal dichalcogenides (TMDs) *MX*_2_ have attracted interest because they display diverse ground states, such as charge density wave and superconductivity. Furthermore, they are considered as candidate type-II Weyl semimetals that could be controlled by tuning the dimensionality or the *d*-electron count of the transition metal *M*, providing a rich avenue to explore novel electronic states^1–5^. Semiconducting behavior is reported with an energy gap of 1 eV for group-6 TMDs with *d*^2^ configuration (*M* = Mo, W; *X* = S, Se, Te). Extremely large magnetoresistances (MR) have been reported in TMDs, including Dirac and Weyl semimetals, where topologically protected states and/or perfect compensation of electron and hole carriers are considered to be the origin of the anomalous MR^6–11^.

MoTe_2_ can exist in one of two polymorphs: a 2H-MoTe_2_ and a distorted 1 T form^12–14^. The 2H-MoTe_2_ phase is semiconducting. In this structure Mo is surrounded by a trigonal prism of Te atoms and the Te-Mo-Te layers are coupled by weak van der Waals forces along the crystalline *c*-axis, making it easy to exfoliate. In contrast, the distorted 1 T or 1 T′ phase is metallic and the Mo atoms are octahedrally surrounded by Te atoms and slightly translated from the center, resulting in zig-zag Mo chains along the *b*-axis. Here, the electrical conduction mainly originates from the bands formed from the Mo *d*-levels^15,16^. The octahedral or trigonal antiprismatic structure of monoclinic 1 T′-MoTe_2_, with space group P2_1_/m illustrated in Fig. S1 of Supplementary Information, possesses inversion symmetry with the center located between Mo atoms in the chain direction^12,17–19^. Non-trivial *Z*_2_-band topology in 1 T′-MoTe_2_ and strong spin-orbit coupling present a unique opportunity for the realization of topological quantum devices^1,2^. The low-*T* orthorhombic T_d_ phase, however, lacks inversion symmetry and the observation of Fermi arcs in the surface state is consistent with a type-II Weyl semimetal phase in MoTe_2_. Recent quantum oscillation measurements, however, revealed disagreement with the bulk Fermi surfaces predicted by band calculations, raising questions about the existence of Weyl points in the low-*T* T_d_ phase of MoTe_2−*x*_^20,21^. Understanding the origin of the extremely large magnetoresistance (XMR) reported in MoTe_2−*x*_, therefore, is important for clarifying the realization of type-II Weyl semimetals as well as its possible technological applications^22,23^.

Here we report the effects of pressure on large magnetoresistance and the Hall and longitudinal conductivities in a single crystal of the transition-metal dichalcogenide MoTe_2−*x*_. Applied pressure suppresses the 1 T′-to-T_d_ phase transition temperature (T*) from 210 K at 1 bar to 58 K at 1.1 GPa and there is no signature of T* at 1.4 GPa, indicating that a *T* = 0 K structural quantum phase transition occurs at a critical pressure *P*_*c*_ between 1.1 and 1.4 GPa. Concomitant with the suppression of T*, the superconducting transition temperature (*T*_*c*_) sharply increases with pressure, from 0.4 K at 0.4 GPa to 2.8 K at 1.4 GPa near *P*_*c*_. On the other hand, *T*_*c*_ gradually increases in the high-pressure 1 T′ phase (*P* > *P*_*c*_) and the Meissner fraction becomes significant, indicating that superconductivity is of bulk nature in the T_d_ phase of MoTe_2_. Large MR observed at 1 bar is strongly suppressed with increasing pressure and is almost saturated at 100% for *P* > *P*_*c*_. The dependence on magnetic field of the Hall and longitudinal conductivities show that a pair of electron and hole bands are dominant in the T_d_ phase, but another pair of electron and hole bands are additionally required in the 1 T′ phase, indicating a subtle change in the Fermi surface topology at *P*_c_. The MR peaks sharply at a characteristic hole-to-electron concentration ratio (*n*_*c*_ ≈ 0.82) and is suppressed as the ratio moves away from *n*_*c*_ in the T_d_ phase. These results establish the comprehensive temperature-pressure phase diagram of MoTe_2_ and underscore that the origin of its MR is the balanced electron-hole carrier concentration.

## Results

**Figure 1:**
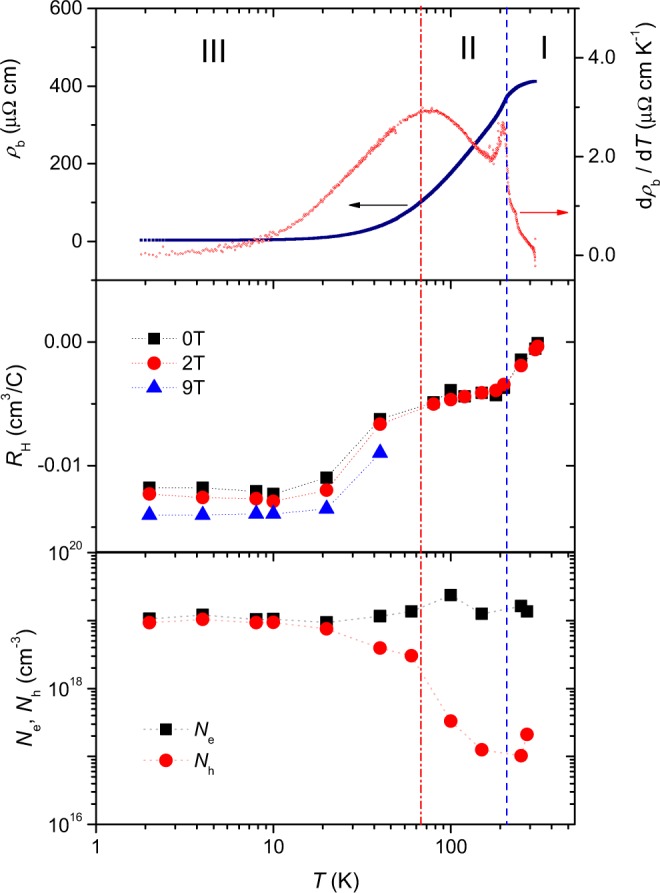
Transport property of MoTe_2−*x*_ as a function of temperature. Upper panel: Electrical resistivity for current along the *b*-axis (=*ρ*_b_) is shown on a semilogarithmic scale on the left ordinate. The first derivative of *ρ*_b_ with respect to temperature, *dρ*_b_/*dT*, is displayed on the right ordinate. There are two features in *dρ*_b_/*dT*: a sharp peak at 210 K, marked by the dashed line, and a broad peak at around 60 K, marked by the dash-dotted line. Regimes I, II, and III are demarcated by the peaks in *dρ*_b_/*dT*. Middle panel: The Hall coefficient *R*_H_ (the ratio of transverse resistivity dividied by applied field) is shown as a function of temperature, where squares, circles, and triangles correspond to *R*_H_ for fields of 0, 2, and 9 T, respectively. Lower panel: Electron (*N*_e_) and hole (*N*_h_) carrier densities are plotted as a function of temperature. Hall and longitudinal resistivity measurements were used to estimate the carrier density, as detailed in Fig. S2 of the Supplementary Information.

The upper panel of Fig. 1 representatively displays the electrical resistivity (*ρ*_b_) of MoTe_2−*x*_ on a semi-logarithmic scale for current applied along the Mo-chain direction (*b*-axis) at ambient pressure. *ρ*_b_ decreases with decreasing temperature, characteristic of metallic behavior. The residual resistivity ratio (RRR) is 147, which is higher than 35 reported in ref.^16^, but is lower than 2000, which was obtained by the authors of ref.^20^. The amount of Te vacancies *x* has been shown to be pertinent to the RRR of MoTe_2−*x*_ as well as *T*_*c*_, e.g., RRR is 13 and *T*_*c*_ is 2.1 K for *x* = 0.13 (ref.^24^). Energy-dispersive spectroscopy (EDS) results show that the Te deficiency is *x* = 0.08 for the crystals that we study in this communication. A kink-like feature in *ρ*_b_ that appears near 210 K (= T*) is consistent with a structural phase transition from the monoclinic (1 T′) to orthorhombic phase (T_d_), where the monoclinic angle β changes from 93.55° to 90°^25,26^. A sharp peak in *dρ*_b_/*dT* (red line), as shown in the right ordinate, clearly demarcates the structural phase transition near 210 K.

The Hall coefficient of MoTe_2−*x*_ at ambient pressure (*R*_H_) is plotted as a function of temperature for fields of 0, 2, and 9 T in the middle panel of Fig. 1, where the magnetic field is applied along the *c*-axis and the Hall voltage is measured across the *a*-axis. *R*_H_ at room temperature is −3.65 × 10^−4^ cm^3^/C, indicating that electron carriers predominantly contribute to the transport. Analyses of the field dependence of the longitudinal (*ρ*_*xx*_) and transverse resistivity (*ρ*_*xy*_) show that the electron and hole carrier densities at room temperature are 1.36 × 10^19^ cm^−3^ and 2.1 × 10^17^ cm^−3^, respectively (see Fig. S2, Supplementary Information). The absolute value of *R*_H_ gradually increases with decreasing temperature and plateaus below 210 K, the critical temperature T* where MoTe_2−*x*_ goes through a structural phase transition from 1 T′ to T_d_ phases. With further decrease of temperature, *R*_H_ starts to decrease again below 60 K, the temperature at which a broad peak in *dρ*_b_/*dT* appears, and the electron and hole carrier densities, *N*_*e*_ and *N*_h_, respectively, become similar. We note that the strong change of the carrier density, the nearly compensated electron-hole carriers (*N*_e_/*N*_h_ ≈ 1) at low temperatures, and a broad peak in the specific heat divided by temperature (*C/T*) are consistent with a Lifshitz-like transition in the candidate Weyl semimetal T_d_-phase of MoTe_2−*x*_^24,27–30^.

**Figure 2:**
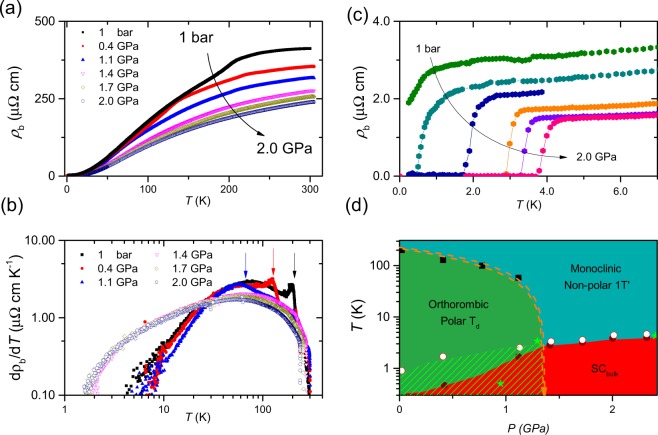
Pressure evolution of electrical resistivity and temperature-pressure phase diagram (**a**) Pressure evolution of the electrical resistivity of MoTe_2−*x*_ for current along the *b*-axis, *ρ*_b_, plotted as a function of temperature. (**b**) First temperature derivative of the resistivity, *dρ*_b_/*dT*, plotted versus temperature for several representative pressures. Arrows mark the peak in the slope where the monoclinic-to-orthorhombic structural phase transition occurs. (**c**) Electrical resistivity of MoTe_2−*x*_ near the superconducting phase transition temperature *T*_c_. (**d**) Temperature-pressure phase diagram of MoTe_2−*x*_, where T_d_ and 1 T′ represent the orthorhombic and monoclinic structure, respectively. Squares and circles represent the structural transition temperature T* and SC transition temperature *T*_c_, respectively. Open and closed circles were determined from the onset and zero-resistance temperature, respectively, of the SC phase transition.

Figure 2(a) shows the pressure evolution of the electrical resistivity of MoTe_2−*x*_ as a function of temperature. *ρ*_b_ at room temperature progressively decreases with increasing pressure at a rate of −7.9 ± 0.6 µΩ cm/GPa because of the increased overlap between adjacent Mo orbitals. The structural transition temperature T*, which was assigned to the peak in *dρ*/*dT* as a function of *T*, decreases to 58 K at 1.1 GPa and is completely suppressed to 0 K below 1.4 GPa, indicating that a *T* = 0 K structural quantum phase transition (=*P*_*c*_) is located between 1.1 and 1.4 GPa. The temperature dependence of *dρ*/*dT* also shows a clear demarcation across *P*_*c*_: the resistivity slope is strongly suppressed in the low-pressure regime (*P* < *P*_*c*_), but it is gradually suppressed in the high-pressure regime (*P* > *P*_*c*_). The low-*T* resistivity slopes collapse on top of each other in the two separate regimes, reflecting two different crystalline structures of T_d_ and 1 T′ in the low and high-pressure regime, respectively. The critical pressure *P*_*c*_ observed in this work is similar to that in ref.^31^ but is much lower than that in refs^16,32^, which may be ascribed to the degree of Te vacancies that is relevant to *T*_*c*_ value at ambient pressure^24^. More systematic study, however, is necessary to understand the precise relationship between *T*_*c*_ and *P*_*c*_.

Being commensurate with the suppression of T*, a superconducting (SC) state is induced and enhanced with increasing pressure, as shown in Fig. 2(c). The onset temperature of the SC phase transition, *T*_c,on_, is 0.9 K at ambient pressure and gradually increases with pressure, developing a smooth dome shape over the whole pressure range. In contrast, a zero-resistance SC state is not observed down to 0.25 K at ambient pressure but appears at 0.47 K and 0.4 GPa. As pressure increases further, the transition temperature *T*_c_ sharply increases and becomes similar to *T*_c,on_ above *P*_c_. As summarized in the temperature-pressure phase diagram in Fig. 2(d), the large difference between *T*_c,on_ and *T*_c_ in the T_d_ phase disappears in the 1 T′ monoclinic phase, indicating that the pressure-induced SC state of MoTe_2−*x*_ in the T_d_ phase is not a bulk property. Substantial differences between *T*_c,on_ and *T*_c_ have been often observed when another competing phase coexists with superconductivity, for example in the quantum critical compound CeRhIn_5_^33^ or in BiS_2_-based superconductors^34^. Supporting this conclusion, the Meissner effect in the magnetic susceptibility is negligible at low pressures, but it is significant for pressures higher than *P*_c_ (see Fig. S3, Supplementary Information). We note that recent neutron scattering experiments reported coexistence of T_d_ and 1 T’ phases at moderate pressures below *P*_*c*_ (ref.^35^).

**Figure 3:**
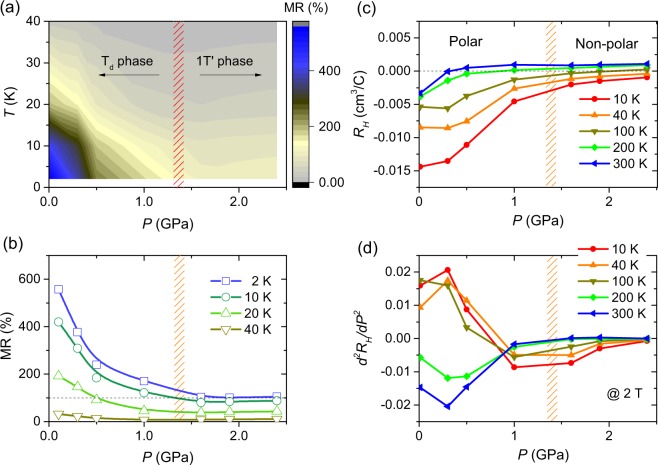
Pressure dependences of MR and Hall coefficients. (**a**) Three-dimensional contour of magnetoresistance (MR) of MoTe_2_ at 9 T plotted in the temperature-pressure plane. Blue color indicates the highest MR. The hashed area marks the critical pressure *P*_*c*_, where the T_d_-to-1T′ structural transition occurs. (**b**) Isothermal magnetoresistance at 9 T plotted as a function of pressure for several representative temperatures. The MR saturates at pressures above *P*_c_. (**c**) Hall coefficients at 2 T plotted as a function of pressure for several representative temperatures. (**d**) The second pressure derivatives of the Hall coefficients from (**c**) plotted as a function of pressure at 10, 40, 100, 200, and 300 K. There are disparate behaviors across *P*_*c*_.

Figure 3(a) shows a MR contour plot of MoTe_2−*x*_ at 9 T in the temperature-pressure plane. The large MR centered on the low-pressure and low-temperature regime is gradually suppressed with increasing temperature and pressure. Figure 3(b) shows the pressure dependence of the isothermal MR at 9 T for representative temperatures of 2, 10, 20, and 40 K. The large MR at 2 K is rapidly suppressed with increasing pressure and is almost saturated at 100% near *P*_c_. The pressure dependence of MR at higher temperatures is similar to that at 2 K, showing a disparate behavior across *P*_*c*_: rapid suppression with pressure in the T_d_ phase, but saturation or slight upturn in the 1 T′ phase. As shown in Fig. 3(c), the pressure dependence of the Hall coefficient (*R*_H_) also shows different behaviors across *P*_*c*_. At low pressures, *R*_H_ is negative and becomes more prominent as temperature falls, indicating that the contribution from electron carriers is more prominent than that from hole carriers. Initially, the absolute value of *R*_H_ decreases rapidly with pressure but is almost flat or changes gradually at higher pressures (*P* > *P*_*c*_) – see Fig. S4, Supplementary Information. The pressure dependence of *R*_H_ is more distinctive in the second pressure derivative (d^2^*R*_H_/d*P*^2^), as shown in Fig. 3(d). In the low-pressure regime (*P* < *P*_*c*_), curvature of the Hall coefficient is negative at high temperatures in the 1 T′ phase and is positive at low temperatures in the T_d_ phase; whereas, in the high-pressure regime (*P* > *P*_*c*_), the curvature is almost negligible for both temperature regimes of the 1 T′ phase.

## Discussion

**Figure 4:**
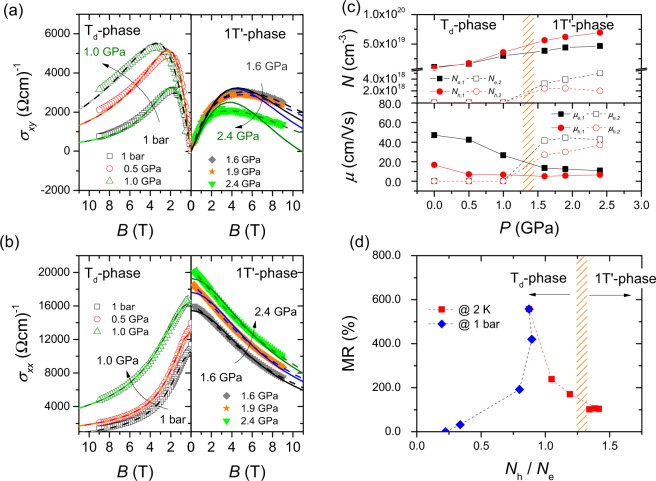
Pressure dependences of Hall and longitudinal conductivities of MoTe_2_ at 2 K are plotted as a function of magnetic field in (**a**,**b**), respectively. Conductivities for selected pressures in the T_d_ and 1 T′ phases are representatively shown in the left and right panels, respectively. Effective two- and four-band models are simulated to understand the field dependence of the conductivities and plotted as solid and dotted lines, respectively. (**c**) Top (bottom) panel: Electron and hole carrier densities (mobilities) obtained from the four-band simulations are plotted as a function of pressure. *N*_e*i*_ (*μ*_ei_) and *N*_h*i*_ (*μ*_hi_) represent, respectively, electron and hole carrier densities (mobilities), and the subscript *i* is the band index. The hashed area represents the critical pressure at low temperatures where the T_d_-to-1T′ transition occurs. (**d**) MR plotted as a function of the hole-electron concentration ratio, *N*_h_/*N*_e_. Red squares represent the pressure-tuned data at 2 K, while blue diamonds represent the temperature-tuned data at ambient pressure.

The pressure-induced suppression of the large MR and the disparate curvature of the Hall coefficient *R*_H_ across *P*_*c*_ indicate that electronic structure is important to the anomalous MR behavior in the T_d_ phase of MoTe_2_. In order to probe the pressure-driven change in electronic structure, the dependence on magnetic field of the transverse (*σ*_*xy*_) and longitudinal (*σ*_*xx*_) conductivities was measured and analyzed using various multi-band models, as shown in Fig. 4(a,b), respectively. For clarity, low-pressure data in the T_d_ phase are plotted in the left panel and high-pressure data in the 1 T′ phase are in the right panel. A two-band isotropic model, as described by the solid lines, reasonably explains the Hall and transverse conductivities in the T_d_ phase (*P* < *P*_c_). At higher pressures (*P* > *P*_c_), however, the field dependence of *σ*_*xy*_ deviates significantly from the two-band model, indicating a change in the electronic structure in the 1 T′ phase. As shown in Fig. S5 in the Supplementary Information, simulations of three-band models do not fit *σ*_*xy*_ in the high-pressure 1 T′ phase either. Dashed lines in Fig. 4(a,b) are simulations of a four-band model, which reasonably explains both *σ*_*xy*_ and *σ*_*xx*_, indicating that the effective two bands in the T_d_ phase evolve into four bands in the 1 T′ phase.

Figure 4(c) plots the pressure evolution of the carrier concentrations of MoTe_2_ at 2 K, which are obtained from simulations of the effective four-band model^36^:

1 $${\sigma }_{xy}=[\sum _{i=1}^{2}\,\frac{{n}_{e,i}{\mu }_{e,i}^{2}}{1+{({\mu }_{e,i}{\mu }_{0}H)}^{2}\,}-\sum _{j=1}^{2}\,\frac{{n}_{h,j}{\mu }_{h,j}^{2}}{1+{({\mu }_{h,j}{\mu }_{0}H)}^{2}}]e{\mu }_{0}H$$

2 $${\sigma }_{xx}=e[\sum _{i=1}^{2}\,\frac{{n}_{e,i}{\mu }_{e,i}}{1+{({\mu }_{e,i}{\mu }_{0}H)}^{2}}+\sum _{j=1}^{2}\,\frac{{n}_{h,j}{\mu }_{h,j}}{1+{({\mu }_{h,j}{\mu }_{0}H)}^{2}}]$$

where $${n}_{e,i}$$$$({\mu }_{e,i})$$ and $${n}_{h,j}$$$$({\mu }_{h,j})$$ are *i*th electron and *j*th hole carrier densities (mobilities), respectively. In the T_d_ phase (*P* < *P*_*c*_), it is effectively a two-band model because the carrier densities for the second electron (*e*_2_) and hole (*h*_2_) bands are negligible. At ambient pressure and 2 K, the carrier densities are 1.06 × 10^19^ and 0.93 × 10^19^ cm^−3^ for the first electron (*e*_1_) and hole (*h*_1_) bands, respectively, showing that they almost compensate each other. At 0.5 GPa, both *e*_1_ and *h*_1_ carrier densities are enhanced to 1.62 × 10^19^ and 1.70 × 10^19^ cm^−1^, respectively, but the carrier density ratio between *e*_1_ and *h*_1_ is reversed. With further increasing pressure, up to the highest measured pressure of 2.4 GPa, both carrier densities increase gradually. The additional two bands, in contrast, are not negligible anymore in the high-pressure 1 T′ phase (*P* > *P*_*c*_). The carrier concentrations are 3.21 × 10^18^ and 2.38 × 10^18^ cm^−3^ at 1.6 GPa for the *e*_2_ and *h*_2_ bands, respectively, which accounts for 8.4% of *N*_e1_ and 4.2% of *N*_h1_ at this pressure. The evolution of the carrier densities is plotted in the pressure-temperature plane in Fig. S6, Supplementary Information. As shown in the bottom panel of Fig. 4(c), the mobilities of *e*_1_ and *h*_1_ bands gradually decrease with pressure, while those of the minority bands (*e*_2_ and *h*_2_) sharply increase above *P*_*c*_ and become larger than the mobilities of the major bands.

**Figure 5:**
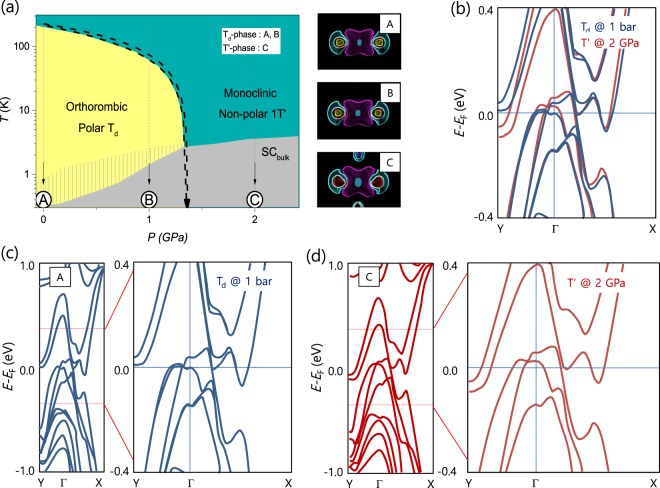
Electronic structure of the T_d_ and pressure-induced 1 T′ phases. (**a**) Schematic *T-P* phase diagram of MoTe_2_. Electronic structure calculations are performed for (A), (B), and (C), which are marked by the arrows, and their Fermi surfaces are plotted at the right of the panel. (**b**) Representative electronic structures are comparatively plotted along the Y-Γ-X direction at 1 bar (blue) and 2 GPa (red). Electronic structures at 1 bar (**c**) and 2 GPa (**d**) are magnified near the Fermi level along the Y-Γ-X direction.

Electronic band structure calculations in the T_d_ and pressure-induced 1 T′ phases were performed via the WIEN2k package with the full-potential linearized augmented plane-wave method and spin-orbit coupling. As shown in Fig. 5, two major pockets (one electron and one hole) and two minor pockets (one electron and one hole) exist along the X-Γ-Y direction in the Brillouin zone. The Fermi surfaces of the four pockets are enlarged when the structural change occurs from the T_d_ to the 1 T′ phase. In the T_d_ phase (*P* < *P*_c_), two major pockets contribute to the anomalously large MR and other physical properties, while the two minor pockets become important in the 1 T′ phase (*P* > *P*_c_). These results are consistent with the analysis of the pressure dependence of the Hall and longitudinal conductivities, underlining that the change in electronic structure plays a critical role in producing the anomalously large MR in MoTe_2_. Supporting this conclusion, the MR peaks sharply at a characteristic electron-hole concentration ratio, i.e., *N*_h_/*N*_e_ = 0.83, as shown in Fig. 4(d). In the T_d_ phase, the MR is rapidly suppressed as the electron-hole concentration ratio moves away from this critical value. In the pressure-induced 1 T′ phase, the ratio slightly changes with pressure and the MR is almost constant (~100%). The Saturation of MR above *P*_*c*_ seems related to the saturation of *N*_h_/*N*_e_ (~1.4) in 1 T′ phase (*P* > *P*_c_). Even though the difference between the major electron and hole carriers gradually increases with increasing pressure, the total carrier concentration ratio is almost saturated due to the contribution from the minor electron and hole carrier, leading to the saturation of MR in 1 T′ phase. We note that the critical ratio deviates from the equal electron-hole concentration point because of different hole and electron mobilities, showing that subtleties of the Fermi surface topology are important in understanding the large MR in MoTe_2_.

The possibility of realizing topological superconductivity in the low-*T* T_d_ phase of MoTe_2_ has been raised. Recent muon-spin relaxation/rotation measurements claimed a topologically non-trivial s^+−^ gap symmetry in the pressure-induced SC phase^32^. However, Takahashi *et al*. pointed out an anticorrelation between superconductivity and the topological T_d_ phase^31^. These contradicting claims stem from controversy about the phase diagrams, especially with respect to the pressure evolution of the T_d_-to-1T′ structural transition^16^. Our systematic transport study under pressure, as shown in Fig. 2(d), resolves this controversy and establishes the comprehensive phase diagram, showing that the structural phase transition is suppressed to 0 K between 1.1 and 1.4 GPa. Ac magnetic susceptibility measurements under pressure revealed that the SC Meissner fraction in the T_d_ phase is negligible but becomes significant in the 1 T′ phase (see Fig. S3, Supplementary Information). The large difference between *T*_c,on_ and *T*_c_ in the T_d_ phase is consistent with the negligible SC volume fraction, underlining that bulk superconductivity in MoTe_2_ exists in the non-polar 1 T′ phase, while there is a possibility of non-bulk superconductivity in the polar T_d_ phase. Surface sensitive studies under pressure will be important for probing the realization of topological superconductivity in the low-pressure T_d_ phase.

## Conclusion

We have reported the dependence on pressure of the large magnetoresistance (MR) and superconductivity in the candidate type-II Weyl semimetal MoTe_2_. With increasing pressure, the monoclinic-to-orthorhombic (T′-to-T_d_) structural phase transition temperature gradually decreases and extrapolates to 0 K at *P*_*c*_ between 1.1 and 1.4 GPa. Supporting the existence of *P*_*c*_, the first temperature derivative of electrical resistivity displays disparate scaling behaviors across *P*_*c*_ and the second pressure derivative of the Hall coefficient becomes saturated above *P*_*c*_. The magnetic field dependence of the Hall and longitudinal conductivities are analyzed by an effective four-band model, where the carrier density ratio between the hole and electron types (*N*_h_/*N*_e_) grows with pressure and becomes saturated above *P*_*c*_. The large non-saturating MR is peaked when *N*_h_/*N*_e_ is 0.83 and is suppressed rapidly as the ratio deviates from the critical value in the T_d_ phase, showing that the origin of the anomalously large MR is a consequence of balancing electron-hole contributions. The small SC volume fraction in the T_d_ phase indicates that bulk superconductivity resides in 1 T′ phase, and thus surface sensitive measurements under pressure are required to properly understand the possible topological nature of its superconductivity.

## Methods

MoTe_2−*x*_ single crystals were synthesized by a NaCl-flux method, for which the detailed methods are described elsewhere^2^. A clamp-type hybrid cell with Daphne 7373 as a pressure transmitting medium was used up to a pressure of 2.5 GPa. A sharp resistivity drop at the superconducting transition temperature of Pb, which was used to determine the pressure inside the cell, is indicative of a quasi-hydrostatic pressure environment^38,39^. A conventional six-probe technique was used to measure the electrical resistivity and Hall effect under an external magnetic field^37^. A Quantum Design Physical Properties Measurement System (PPMS) and a ^3^He Heliox system (Oxford Inst. Nanotechnology) are used to regulate temperature down to 1.8 K and 0.25 K, respectively.

## Electronic supplementary material

Supplementary Information
