# Supplementary material for: Origin of extremely large magnetoresistance in the candidate type-II Weyl semimetal MoTe2−x
Source: Sci Rep. 2018 Sep 17;8:13937. doi: 10.1038/s41598-018-32387-1 (PMC6141610; doi:10.1038/s41598-018-32387-1)
Supplement: Supplementary file 1 — Supplementary Information [file 41598_2018_32387_MOESM1_ESM.pdf]

Supplementary Information for

Origin of extremely large magnetoresistance in the candidate type-II Weyl semimetal $MoTe_{2-x}$

Sangyun Lee[1], Jaekyung Jang[2], Sung-Il Kim[1], Soon-Gil Jung[1], Jihyun Kim[1], Suyeon Cho[3,4], Sung Wng Kim[5], Joo Yull Rhee[2], Kee-Su Park[1], and Tuson Park[1*]

Supplementary Information includes experimental methods, simulations of the Hall and longitudinal conductivities from various multi-band models and electronic structure calculations of $MoTe_2$ via the WIEN2K package. Supplementary figures are included to support the main text.

Figure S1 – Crystal structure of the 1T′ and T_d phase of $MoTe_2$.

Figure S2 – Hall and longitudinal conductivities of $MoTe_2$ at 1 bar.

Figure S3 – Ac magnetic susceptibility of $MoTe_2$ under pressure.

Figure S4 - 3D plot of the Hall coefficient of $MoTe_2$ versus temperature and pressure.

Figure S5 – Simulations using various multi-band models.

Figure S6 – 3D plot of the carrier densities versus temperature and pressure.

Figure S7 –Total energies of $MoTe_2$ in the T_d and 1T′ phases.

Two-band isotropic model

Hall and longitudinal conductivities as a function of external magnetic field are derived as $\sigma_{xx} = \frac{\rho_{xx}}{(\rho_{xy}^2+\rho_{xx}^2)}$ and $\sigma_{xy} = \frac{-\rho_{xy}}{(\rho_{xy}^2+\rho_{xx}^2)}$. Generally, a two-band isotropic model can successfully describe the transport properties of a number of materials with electron and hole bands crossing the Fermi level[1-8]. The conductivity tensor is written as[3]:

$$\hat{\sigma}_2 = e\left[\frac{n_e\mu_e}{1+i\mu_e\mu_0 H} + \frac{n_h\mu_h}{1-i\mu_h\mu_0 H}\right] \quad (1),$$

where $n_e(\mu_e)$ and $n_{\mathrm{h}}(\mu_h)$ are electron and hole carrier densities (mobilities), respectively. The subscripted index 2 indicates that it is derived via the two-band isotropic model. Hall and longitudinal conductivities are derived as follows[1-4]:

$$\sigma_{xy,2} = \left[n_e\mu_e^2\frac{1}{1+(\mu_e\mu_0 H)^2} - n_h\mu_h^2\frac{1}{1+(\mu_h\mu_0 H)^2}\right]e\mu_0 H \quad (2),$$

$$\sigma_{xx,2} = e\left[\frac{n_e\mu_e}{1+(\mu_e\mu_0 H)^2} + \frac{n_h\mu_h}{1+(\mu_h\mu_0 H)^2}\right] \quad (3).$$

Figure S2 shows the Hall resistivity ($1/\sigma_{xy}$) at 1 bar for several representative temperatures and a comparison with simulations of the two-band isotropic model. The good agreement between experiments and simulations indicates that the transport properties of $MoTe_2$ at 1 bar can be described by a single pair of electron and hole bands.

Extended multi-band isotropic model

The conductivity tensor of the two-band model can be extended to a multi-band isotropic model with additional terms[1]:

$$\hat{\sigma}_m = e\left[\sum_{i=1}^{p} \frac{n_{e,i}\mu_{e,i}}{1+i\mu_{e,i}\mu_0 H} + \sum_{j=1}^{q} \frac{n_{h,j}\mu_{h,j}}{1-i\mu_{h,j}\mu_0 H}\right] \quad (4),$$

where $n_{e,i}(\mu_{e,i})$ and $n_{h,j}(\mu_{h,j})$ are ith electron and jth hole carrier densities (mobilities), respectively. The subscripted index m indicates that it is derived via an m-band isotropic model. The number of electron and hole bands are p and q, respectively. Therefore, m, p and $q \in \mathbb{N}$, and $m = p + q$. Hall and longitudinal conductivities can be written as follows:

$$\sigma_{xy,m} = \left[\sum_{i=1}^{p} \frac{n_{e,i}\mu_{e,i}^2}{1+\left(\mu_{e,i}\mu_0 H\right)^2} - \sum_{j=1}^{q} \frac{n_{h,j}\mu_{h,j}^2}{1+\left(\mu_{h,j}\mu_0 H\right)^2}\right] e\mu_0 H \quad (5),$$

$$\sigma_{xx,m} = e\left[\sum_{i=1}^{p} \frac{n_{e,i}\mu_{e,i}}{1+\left(\mu_{e,i}\mu_0 H\right)^2} + \sum_{j=1}^{q} \frac{n_{h,j}\mu_{h,j}}{1+\left(\mu_{h,j}\mu_0 H\right)^2}\right] \quad (6).$$

Simulations of various multi-band models are compared with the experimental data on $MoTe_2$ at 1.9 GPa, which is in the pressure-induced 1T′ phase. Even though the field dependence of the longitudinal conductivity is explained well by the two-, three-, and four-band models, at least four bands are required to explain the field dependence of the transverse (Hall) conductivity.

Electronic structure calculations

Electronic structure calculations of $MoTe_2$ were performed by using the WIEN2k package[9] implemented with the full-potential linearized augmented plane-wave method. The exchange-correlation functional was chosen to be the generalized-gradient approximation of Perdew, Burke and Ernzerhof[10]. The muffin-tin radii were 2.38 and 2.5 a.u. for Mo and Te atoms, respectively. We used RK_{max} = 7, resulting in ～124 augmented plane-waves for the basis functions. For the self-consistent-field cycle, we generated 1000 k-points in the entire Brillouin zone (BZ), which corresponds to 190 k-points in the irreducible wedge of the BZ. Spin-orbit coupling was included in the calculations. Lattice constants of T_d and 1T′ $MoTe_2$ were adopted from ref. 11 for volume optimization calculations.

Figure S7 compares the pressure dependences of the estimated total energies of $MoTe_2$ in the T_d (black squares) and 1T′ phases (red circles). The total energy is lower in the T_d phase than in the 1T′ phase at pressures lower than 1.3 GPa, indicating the presence of a structural quantum phase transition.

References

1. Luo, Y. *et al.* Hall effect in the extremely large magnetoresistance semimetal WTe_2. *Applied Physics Letters* **107,** 182411 (2015).

2. Zhou, Q., Rhodes, D., Zhang, Q. R., Tang, S., Schonemann, R. & Balicas, L. Hall effect within the colossal magnetoresistive semimetallic state of $MoTe_2$. *Phys. Rev. B* **94,** 121101(R) (2016).

3. Rullier-Albenque, F., Colson, D., Forget, A. & Alloul, H. Hall Effect and Resistivity Study of the Magnetic Transition, Carrier Content, and Fermi-Liquid Behavior in $Ba(Fe_{1-x}Co_x)_2As_2$. *Phys. Rev. Lett.* **103,** 057001 (2009).

4. Rullier-Albenque, F., Colson, D., Forget, A., Thu´ery, P. & Poissonnet, S. Hole and electron contributions to the transport properties of $Ba(Fe_{1-x}Ru_x)_2As_2$ single crystals *Phys. Rev. B* **81,** 224503 (2010).

5. Takahashi, H., Okazaki, R., Yasui, Y. & Terasaki, I. Low-temperature magnetotransport of the narrow-gap semiconductor $FeSb_2$. *Phys. Rev. B* **84,** 205215 (2011).

6. Xia, B. *et al.* Indications of surface-dominated transport in single crystalline nanoflake devices of topological insulator $Bi_{1.5}Sb_{0.5}Te_{1.8}Se_{1.2}$. *Phys. Rev. B* **87,** 085442 (2013).

7. Ali, M. N. *et al.* Large, non-saturating magnetoresistance in WTe_2. *Nature* **514,** 205 (2014).

8. Huang, X. *et al.* Observation of the Chiral-Anomaly-Induced Negative Magnetoresistance in 3D Weyl Semimetal TaAs. *Phys. Rev. X* **5,** 031023 (2015).

9. Blaha, P., Schwarz, K., Madsen, G. K. H., Kvasnicka, D. & Luitz, J. WIEN2K, An Augmented Plane Wave + Local Orbitals Program for Calculating Crystal Properties, Karlheinz Schwarz, Techn. Universit¨at Wien, Wien, Austria (2001).

10. Perdew, J. P., Burke, K. & Ernzerhof, M. Generalized Gradient Approximation Made Simple. *Phys. Rev. Lett.* **77,** 3865 (1996).

11. Dawson, W. G .& Bullett, D. W. Electronic structure and crystallography of $MoTe_2$ and WTe_2. *J. Phys. C: Solid State Phys.* **20** 6159 (1987)

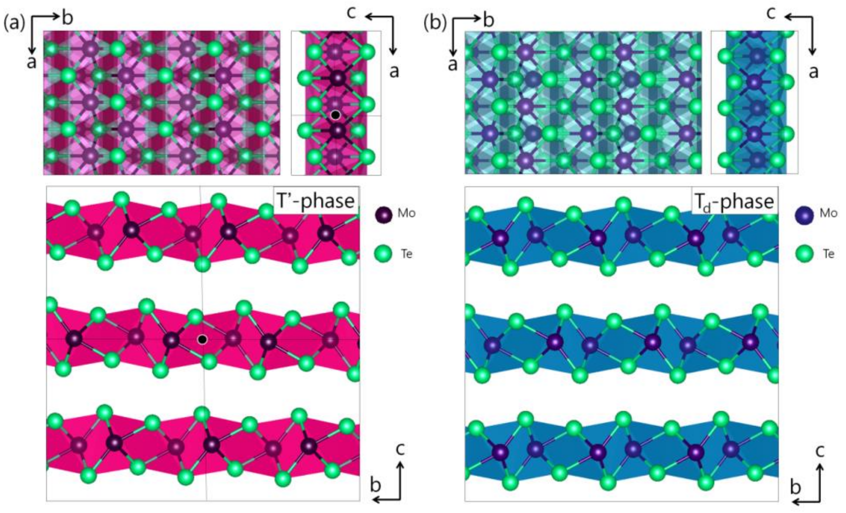

Figure S1. Crystal structure of the 1T′ and T_d phase of $MoTe_2$. (a) Crystal structure of the monoclinic 1T′ phase ($P > P_c$ or $T > T^*$) projected onto the *ab* (upper left panel), *ac* (upper right panel), and *bc* (lower panel) planes. Black circles are inversion centers. (b) Crystal structure of the orthorhombic T_d phase ($P < P_c$ or $T < T^*$) mapped onto the *ab* (upper left panel), *ac* (upper right panel), and *bc* (lower panel) planes. There is no inversion center, showing that the Td phase is noncentrosymmetric.

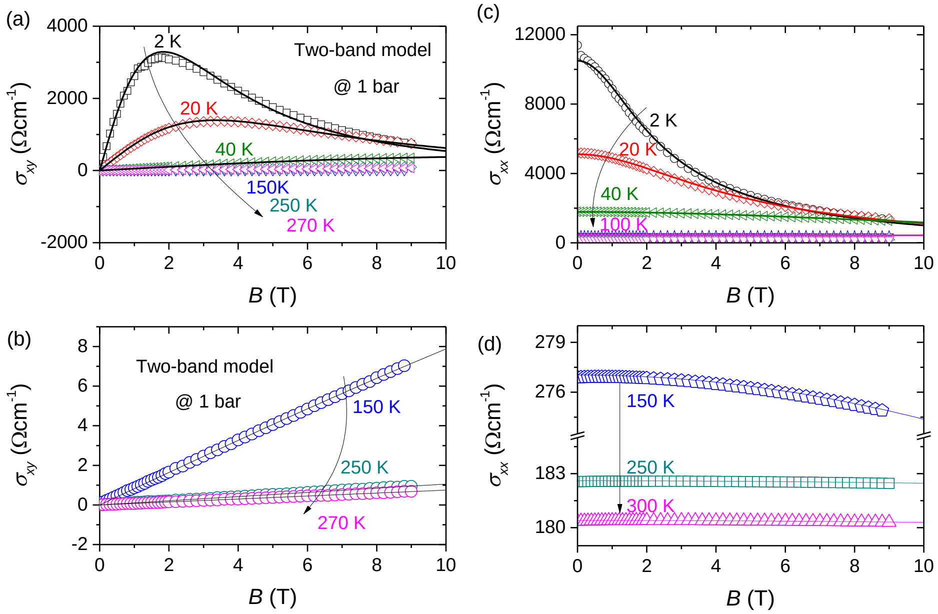

Figure S2. Hall and longitudinal conductivities of $MoTe_2$ at 1 bar. (a) Hall conductivities at 1 bar and 2, 20, 40, 150, 250, and 270 K are plotted against magnetic field. (b) Expanded view of the Hall conductivities at 150, 250, and 270 K shown in (a), illustrating a linear field dependence. (c) Longitudinal conductivities at 1 bar and 2, 20, 40, 150, 250, and 270 K are plotted against magnetic field. (d) Expanded view of the longitudinal conductivities at 150, 250 and 270 K shown in (c). Solid lines are simulations using a two-band isotropic model (see the text for details).

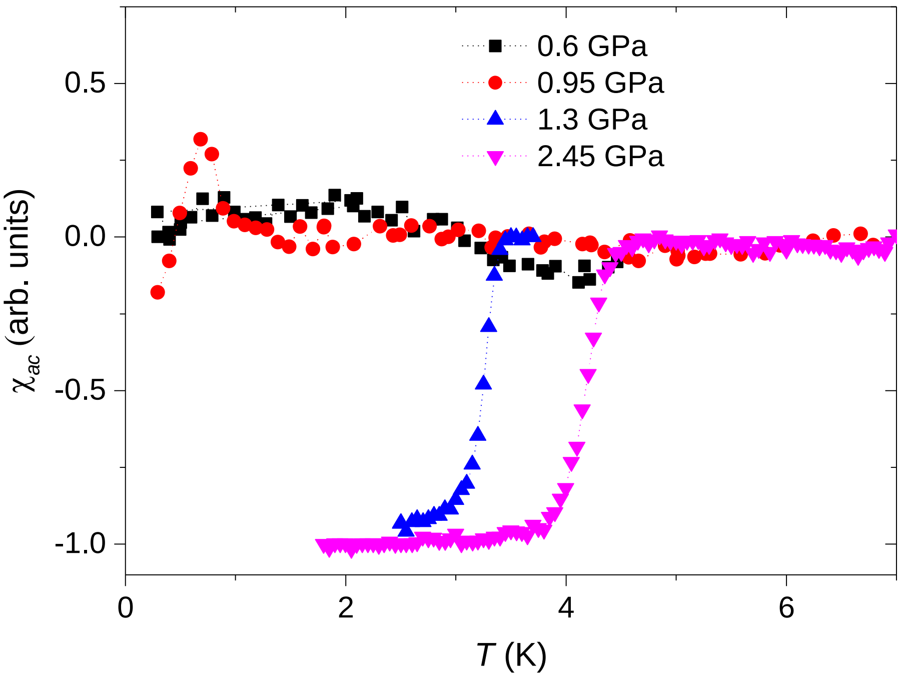

Figure S3. Ac magnetic susceptibility of $MoTe_2$ under pressure. Magnetic susceptibility (χ_{ac}) as a function of temperature at 0.6, 0.95, 1.3 and 2.45 GPa. The superconducting Meissner volume fraction is almost negligible up to 0.95 GPa but is strongly enhanced for pressures higher than 1.3 GPa.

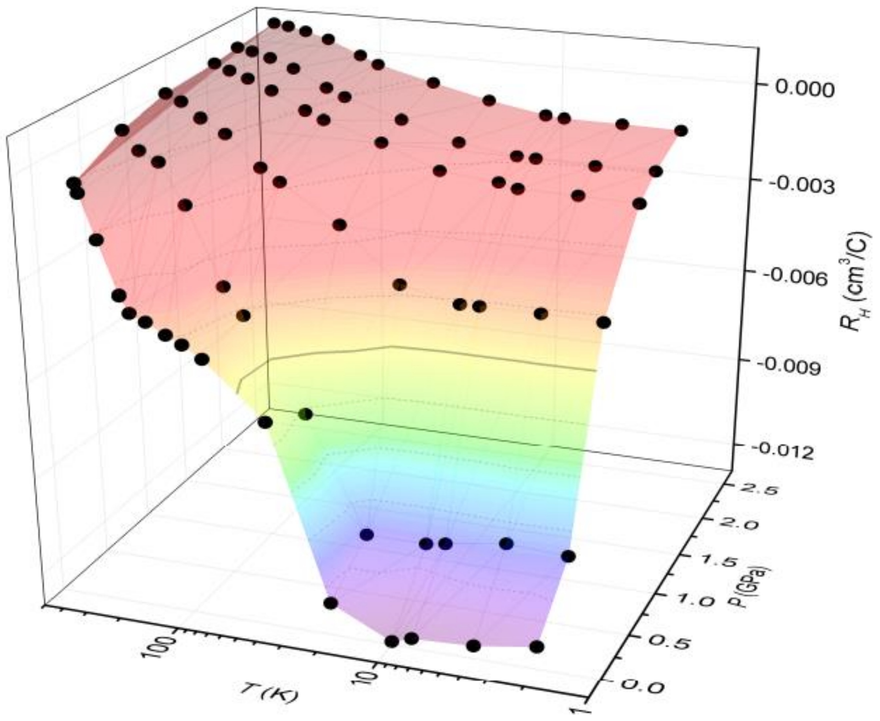

Figure S4. 3D plot of the Hall coefficient of $MoTe_2$ versus temperature and pressure. The absolute value of the Hall coefficient is sharply enhanced below a characteristic temperature of 60 K and a critical pressure P_c (the violet region).

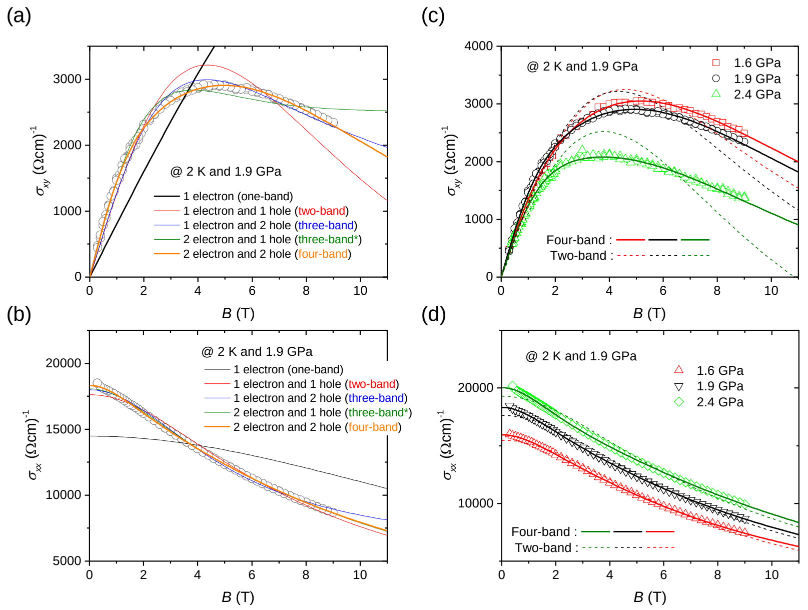

Figure S5. Simulations using various multi-band models. Simulations of the effective one- (black), two- (red), three- (blue), three- (green), and four-band models are compared with Hall and longitudinal electrical conductivities of $MoTe_2$ at 2 K and 1.9 GPa in (a) and (b), respectively. The four-band model best explains both conductivities over the entire pressure range, including the 1T′ phase ($P > P_c$).

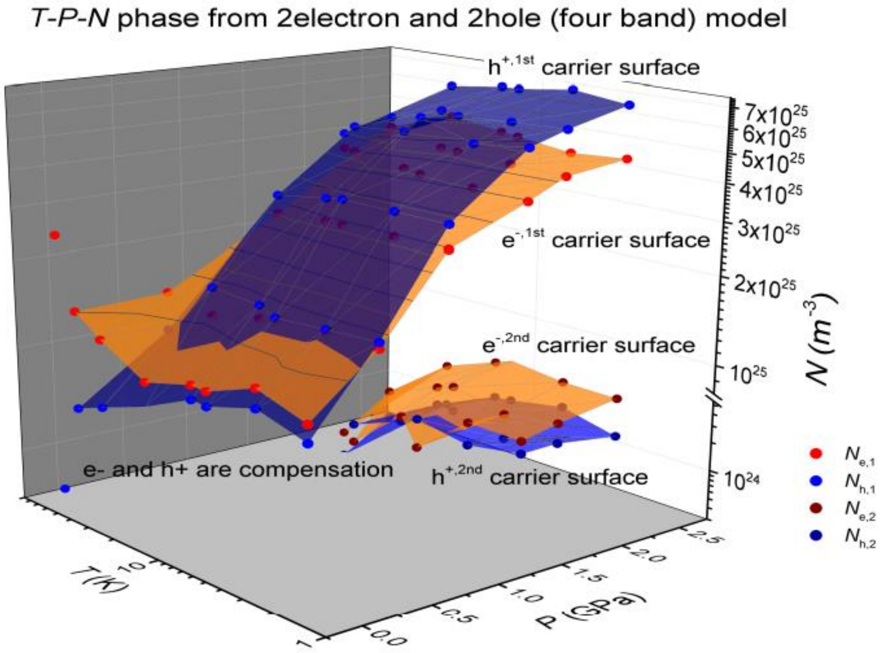

Figure S6. 3D plot of the carrier densities versus temperature and pressure. At 1 bar, the electron and hole carrier concentrations are almost compensated. The hole carrier density increases with increasing pressure and becomes dominant in the 1T′ phase. Small electron and hole bands are introduced to explain the Hall and longitudinal conductivities in the 1T′ phase ($P > P_c$).

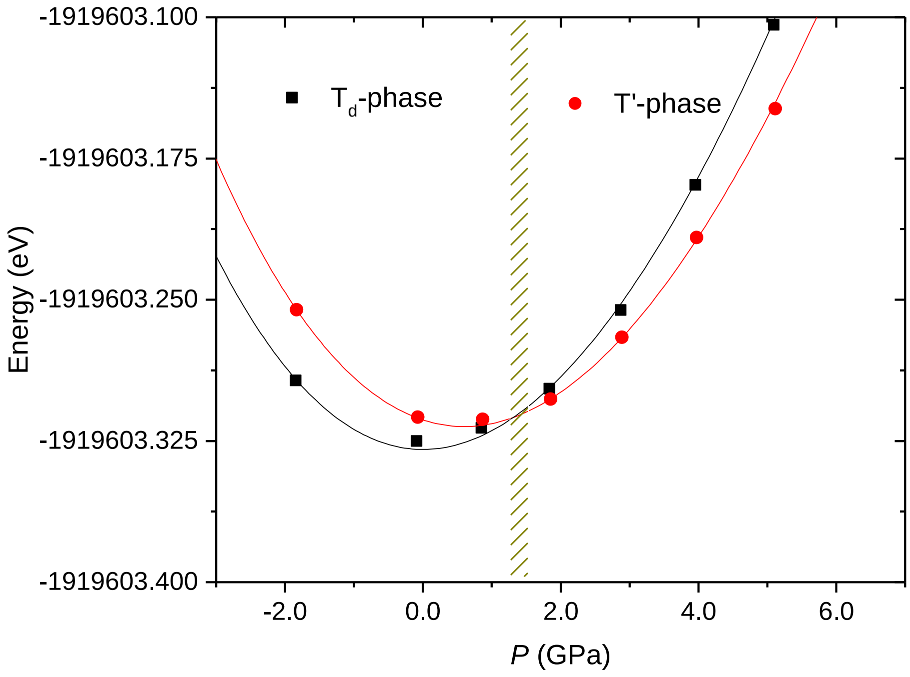

Figure S7. Total energies of $MoTe_2$ in the T_d and 1T′ phases. The total energy of the T_d phase is lower than that of the 1T′ phase below ~1.3 GPa.
